# FOXO3 is targeted by miR-223-3p and promotes osteogenic differentiation of bone marrow mesenchymal stem cells by enhancing autophagy

**DOI:** 10.1007/s13577-020-00421-y

**Published:** 2020-09-13

**Authors:** Cheng Long, Shiqiang Cen, Zhou Zhong, Chang Zhou, Gang Zhong

**Affiliations:** Department of Orthopedic, West China Hospital, Sichuan University, No. 37, Guoxue Alley, Wuhou District, Chengdu, 610041 Sichuan China

**Keywords:** Forkhead Box O3, miR-223-3p, Bone marrow mesenchymal stem cells, Osteogenic differentiation, Autophagy

## Abstract

Mesenchymal stem cells (MSCs) are a promising regenerative medicine. The roles of miRNAs in osteogenic differentiation of bone marrow MSCs (BM-MSCs) remained less reported. Forkhead Box O3 (FOXO3) and alkaline phosphatase (ALP) levels in the BM-MSCs were measured on 3, 7, and 14 days after osteogenic differentiation. After transfection of FOXO3 overexpression plasmids or siFOXO3 into BM-MSCs, factors related to osteogenic differentiation or cell autophagy were determined. Besides, 3-methyladenine or rapamycin, as well as miR-223-3p mimic or inhibitor were applied to further determine the effect of FOXO3 in BM-MSCs. FOXO3 and ALP levels were increased in a time-dependent manner with osteogenic differentiation, supported by Alizarin Red Staining. Furthermore, up-regulated FOXO3 increased levels of ALP and factors related to osteogenic differentiation by increasing levels of autophagy-related factors. FOXO3, targeted by miR-223-3p, reversed the effects of miR-223-3p on factors related to BM-MSC autophagy and osteogenic differentiation. Down-regulated miR-223-3p expression promoted osteogenic differentiation of BM-MSCs by enhancing autophagy via targeting FOXO3, suggesting the potential of miR-223-3p as a therapeutic target for enhancing bone functions.

## Introduction

Mesenchymal stem cells (MSCs) could be isolated from human tissues such as bone marrow, adipose tissue, and birth-related tissues, for example, placenta, umbilical cord, cord blood, or amnion. Multifunctional MSCs have the abilities of self-renewal and differentiation into osteoblasts, chondrocytes, or adipocytes [1]. Bone marrow MSCs (BM-MSCs), in particular, play a pivotal role during bone formation, and have attracted great attention for their ability to induce osteogenic differentiation and their secretion of growth factors and cytokines [2]. In clinical practice, osteogenic differentiation of BM-MSCs has been widely applied to the treatments such as large bone fracture and bone-related tissue engineering [3]. However, abnormal osteogenic differentiation of BM-MSCs was related with the onset and progression of several diseases, such as osteoarthritis (OA) and ankylosing spondylitis (AS) [4, 5]. A deeper understanding of the molecular mechanisms underlying osteogenic differentiation of BM-MSCs has clinical significance for bone disease pathology and clinical treatment.

The previous studies showed that Forkhead Box O3 (FOXO3) plays a critical role in osteogenic differentiation through inducing autophagy [6], and that FOXO3 might be the target of miR-96-5p, miR-629 and miR-149 [7–9]. MiRNAs, which are a class of endogenous RNAs of 19–25 nucleotides in total length, exert post-transcriptional effects on gene expressions, and some of them have been also found to affect osteogenic differentiation of MSCs [10, 11]. The previous study showed that FOXO3 was the target for miR-223-3p [12]. However, the role and regulation mechanisms of FOXO3 in relation to miRNAs in osteogenic differentiation of BM-MSCs still remained to be explored.

Autophagy referred to an evolutionarily conserved catabolic process in which cells deliver cytoplasmic degradation components into lysosomes [13]. Autophagy also acts as pro-survival or pro-death response under physiological or pathological conditions [14, 15]. The previous studies suggested that the activation of autophagy might be related to the promotion of osteogenic differentiation [16–18]. However, the effects of miRNAs, miR-223-3p in particular, on enhancing MSCs autophagy to promote osteogenic differentiation remained to be fully understood. Thus, this study mainly examines the role of miR-223-3p on the autophagy and osteogenic differentiation of MSCs and the relations between autophagy and osteogenic differentiation of BM-MSCs.

## Materials and methods

### Cell culture

Human bone marrow mesenchymal stem cells (BM-MSCs; catalog no. PCS-500-012) were obtained from American Type Culture Collection (ATCC; Manassas, VA, USA) and cultured in low-glucose Dulbecco’s modified Eagle’s medium (DMEM; D5030, Sigma-Aldrich, St Louis, MO, USA) supplemented with 10% fetal bovine serum (FBS; F2442; Sigma-Aldrich, St Louis, MO, USA), 100 U/mL Penicillin (Invitrogen, USA), and 100 μg/mL Streptomycin (Invitrogen, USA).

For osteogenic differentiation, the BM-MSCs were cultured in DMEM (Invitrogen, USA) containing 1% FBS (F2442; Sigma-Aldrich, USA), 200 μM l-glutamine (G7513; Sigma-Aldrich, USA), 10 nM dihydroxyvitamin D3 (D1530; Sigma-Aldrich, USA), 10 mM β-glycerolphosphate (50020; Sigma-Aldrich, USA), 100 nM dexamethasone (D4902; Sigma-Aldrich, USA), and 80 μg/mL ascorbic acid phosphate (A8960; Sigma-Aldrich, USA). And the medium was refreshed every 3–4 days.

To evaluate the effects of Forkhead Box O3 (FOXO3) on the autophagy of BM-MSCs, 100 nM autophagy activator Rapamycin (RAPA; R0395; Sigma-Aldrich, USA) was added into the BM-MSCs transfected with lentivirus carrier of small interfering RNA for FOXO3 (siFOXO3). 5 mmol/L autophagy inhibitor 3-methyladenine (3-MA; M9281; Sigma-Aldrich, USA) was added into BM-MSCs transfected with lentivirus carrier of overexpressed FOXO3 plasmid.

### Lentivirus transfection

Lentivirus carriers for overexpressed FOXO3 were synthesized as previously described [19]. FOXO3 gene was first amplified from template plasmid pcDNA3 FLAG-human FOXO3 (Addgene, Watertown, MA, USA) using polymerase chain reaction (PCR). Then, the product of PCR was cloned into the pENTR11 vector (A10467, Invitrogen, USA) and recombined with Gateway LR Clonase II enzyme mix (11,791,020; Invitrogen, USA) in the Gateway lentiviral expression plasmid pLenti CMV Puro DEST (w118-1) (17,452; Addgene, USA). Small interfering RNA for FOXO3 (siFOXO3; catalog number: A09003) was synthesized by Gene Pharma (Shanghai, China). To evaluate the effect and functions of FOXO3 and miR-223-3p in BM-MSCs, lentivirus carriers for overexpressed FOXO3 and siFOXO3, miR-223-3p mimic, and inhibitor were transfected into BM-MSCs using *Trans*IT-VirusGen^®^ Transfection reagent (MIR6703; Mirus Bio, Madison, WI, USA) in accordance with the producer’s instructions. Before conducting lentivirus transfection, 2 × 10^6^ cells/mL BM-MSCs contained in 2 mL complete medium were seeded into a 6-well plate for 18–24 h. Transfection of lentivirus was performed after the cells reached 80–95% confluence. For lentivirus transfection, after 24 h, cell culture medium was refreshed by 6 μg/mL polybrene and lentivirus carriers.

### Enzyme-linked immunosorbent assay (ELISA)

The BM-MSCs (1 × 10^5^ cells/mL) were seeded into 12-well plates for 24 h at 37 ˚C with 5% CO_2_. Then, alkaline phosphatase (ALP) level in BM-MSCs was measured with ALP ELISA detection kits (EH2618; Fine Biotech, Wuhan, China) on days 3, 7, and 14 after the induction of osteogenic differentiation. OD value was detected and measured at an absorbance of 450 nm using DSX^®^ Ambient System (#65,200; DYNEX Technologies, Chantilly, VA, USA).

### Alizarin red staining

BM-MSCs at a density of 1.5 × 10^4^ cells/mL were cultured into 24-well plates and subjected to Alizarin Red staining on days 0, 3, 7, and 14 after the induction of osteogenic differentiation. In detail, BM-MSCs were first harvested, washed with PBS three times, fixed with 4% paraformaldehyde at room temperature for 20 min, and then washed with distilled water. After washing the cells with PBS for 3–5 times and microscope observation (SW380T, Swift Optical Instruments, Schertz, TX, USA), the BM-MSCs were stained using Alizarin Red S staining kit (SC0223; 3H Biomedical, Uppsala, Sweden) at room temperature for 1 h.

### Prediction on target gene and dual-luciferase reporter assay

Using TargetScan V7.2, we successfully predicted that miR-223-3p was the target for FOXO3b and their potential binding sites, which was further confirmed by dual-luciferase reporter assay.

Plasmids containing FOXO3 3-untranslated regions (3′-UTRs) with miR-223-3p target sites were purchased from Gene pharma and inserted into PMIR-REPORT Luciferase vector (AM5795; Thermo Fisher Scientific, USA). Q5^®^ Site-Directed Mutagenesis Kit (E0554S; New England Biolabs, Ipswich, MA, USA) was used for the mutagenesis of 3′-UTRs.

Verification on the target genes and potential binding sites between miR-223-3p and FOXO3 was performed using dual-luciferase reporter assay. Briefly, reporter plasmids of wild-type or mutated FOXO3 (FOXO3-WT, sequence: 5′-CAAAGCAGACCCUCAAACUGACA-3′; FOXO3-MUT, sequence: 5′-CAAAGCAGACCCUCAGCAGCCGC-3′), miR-223-3p mimic, and inhibitor were transfected into the BM-MSCs (5 × 10^5^ cell/mL) using Lipofectamine 2000 Transfection reagent (Thermo Fisher Scientific, USA) at 37 ˚C. 48 h after the transfection, firefly luciferase activity was detected using dual-luciferase reporter assay system (E1910; Promega, Madison, WI, USA). *Renilla* luciferase activity was used for normalization.

### RNA isolation and quantitative real-time polymerase chain reaction (qRT-PCR)

Total RNA was extracted from the BM-MSCs by Trizol reagent (catalog number: 15596026; Invitrogen, USA) and preserved at 4 ˚C. Following the determination of protein concentration with a Nano Drop 2000 spectrometer (Thermo Fisher Scientific, USA), 1 μg of total RNA was used to synthesize cDNA using a SureScript™ First-strand cDNA Synthesis Kit (QP057; GeneCopoeia, Guangzhou, China). QRT-PCR experiment was conducted with QuantiTect Reverse Transcription kit (205,314; Qiagen, Hilden, Germany) in a 7500 Fast real-time PCR System (Thermo Fisher Scientific, USA) under the conditions as follows: 94 ˚C for 10 min, followed by 40 cycles of 94 ˚C for 30 s, 60 ˚C for 30 s, and 72 ˚C for 40 s. Primer sequences are shown in Table 1. GAPDH and U6 were internal controls. Gene expressions were quantified with $2^{{ - \Delta \Delta C_{{^{{\text{T}}} }} }}$ method [20].

**Table 1:** Primers for qRT-PCR

Gene	Primers
FOXO3	
Forward	5′-TAACTTTGATTCCCTCATCT-3ʹ
Reverse	5′- TAGGTCTTGTGTCAGTTTGA-3ʹ
RUNX2	
Forward	5′-TCTCTGGTTTTTAAATGGTTA-3ʹ
Reverse	5′-CTTGTACCCTCTGTTGTAAAT-3ʹ
OCN	
Forward	5′-ACCGAGACACCATGAGAG-3′
Reverse	5′-CTGGGTCTCTTCACTACCT-3′
Smad4	
Forward	5′-ATGGAGCTCATCCTAGTAAA-3′
Reverse	5′-TGATATGGATTCACACAGAC-3′
Beclin 1	
Forward	5′-GTGGAATGGAATGAGATTA-3′
Reverse	5′-GCTCCTTAGATTTGTCTGTC-3′
P62	
Forward	5′-GTACCAGGACAGCGAGAG-3′
Reverse	5′-ATGTAGATTCGGAAGATGTC-3′
GAPDH	
Forward	5′-TTTTTGGTTTTAGGGTTAGTTAGTA-3′
Reverse	5′-AAAACCTCCTATAATATCCCTCCTC-3′

### Western blot

Protein expressions of genes (Runt-related transcription factor 2, RUNX2; osteocalcin, OCN, SMAD family member 4, Smad4) related to osteogenic differentiation and to autophagy (Beclin1; Light Chain 3, LC3; p62) in the BS-MSCs were measured by Western blot as previously described [21, 22]. RIPA buffer (#9806; Cell Signaling Technology, Danvers, MA, USA) was adopted to extract and lyse proteins from BM-MSCs, and bicinchoninic acid (BCA) protein kit (ab102536, Abcam, Cambridge, UK) was used to quantify the concentration. Sample protein lysates (30 μg) were subsequently electrophoresed by sodium dodecyl sulfate-polyacrylamide gel electrophoresis (SDS-PAGE; P0012A; Beyotime, Shanghai, China) and then transferred onto polyvinylidene fluoride (PVDF) membrane (FFP28; Beyotime, China). The membrane was first blocked with nonfat milk (5%) for 2 h and then was incubated with the following primary antibodies: anti-RUNX2 antibody (ab23981, rabbit, 1:5000, Abcam, UK), anti-OCN antibody (ab93876, rabbit, 1:500, Abcam, UK), anti-Smad4 antibody (ab40759, rabbit, 1:5000, Abcam, UK), anti-Beclin 1 antibody (ab207612, rabbit, 1:2000, Abcam, UK), anti-LC3B antibody (ab48394, rabbit, 1:2000, Abcam, UK), anti-p62 antibody (ab109012, rabbit, 1:10,000, Abcam, UK), and anti-GAPDH antibody (ab181602, rabbit, 1:10,000, Abcam, UK) at 4℃ overnight, with GAPDH as internal control. Next, the membrane was incubated in secondary horseradish peroxidase (HRP)-conjugated antibody goat anti-rabbit IgG H&L (HRP) (1:20,000, AS09602, Agrisera, Vännäs, Sweden) at room temperature for 1 h and washed with tris-buffer saline tween (TBST) for three times. Protein band was collected and analyzed by Amersham ECL Western Blotting Detection kit (RPN2108; Global Life Sciences Solutions, Marlborough, MA, USA). Gray values of the strips were calculated by ImageJ 5.0 (National Institutes of Health, Bethesda, MD, USA).

### Statistical analysis

The data were expressed as mean ± standard deviation (SD). The experiments were performed in triplicate. Statistical analysis was performed using SPSS (ver. 20.0; Chicago, IL, USA). Statistical significance between groups were determined by one-way ANOVA and Student’s *t* test. At least *P* < 0.05 was considered as statistically significant.

## Results

### Increased FOXO3 expression and ALP were observed during osteogenic differentiation

**Fig. 1:**
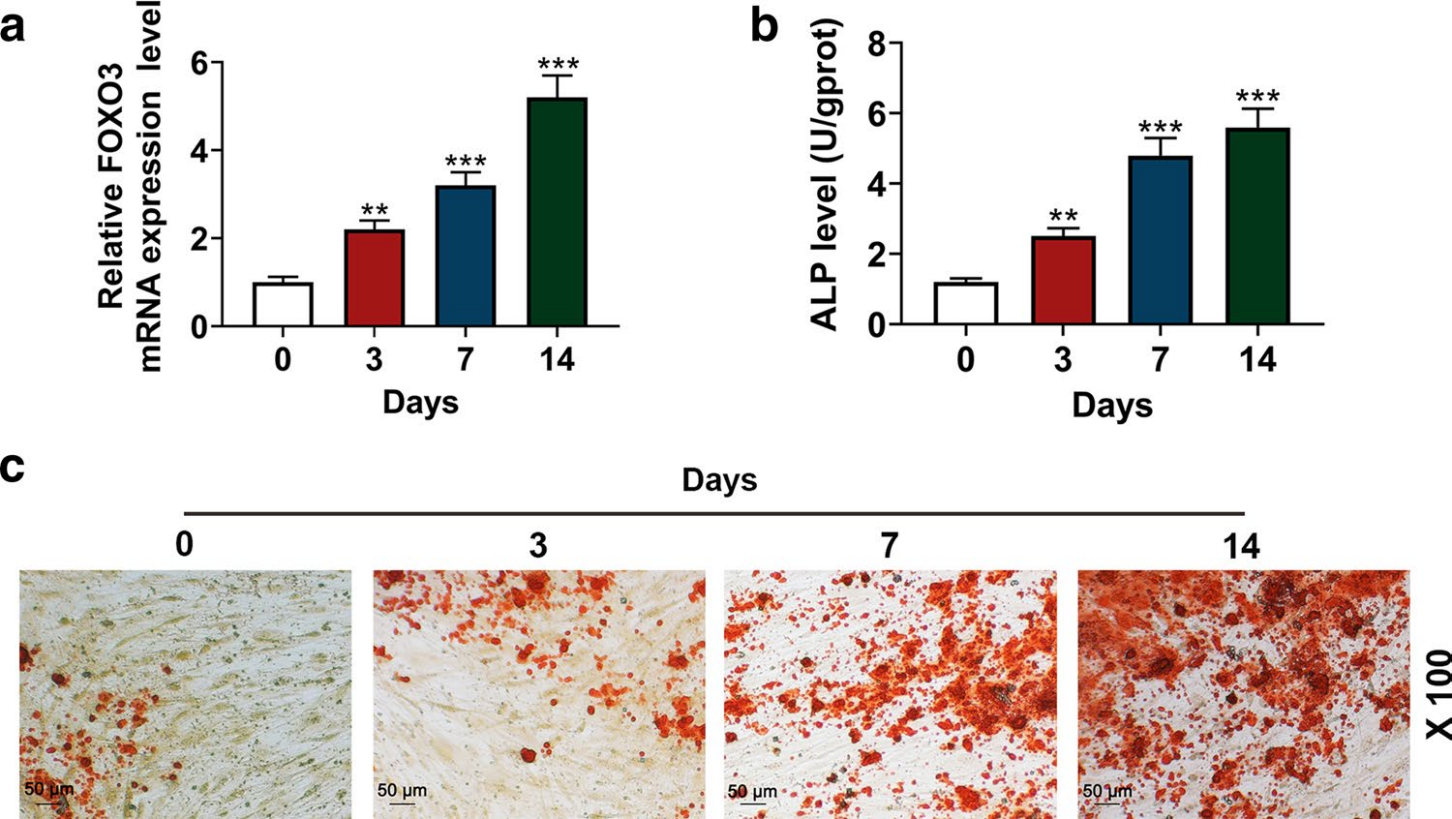
FOXO3 expression and ALP level were increased during osteogenic differentiation. **a** Relative FOXO3 expressions in BM-MSCs on days 0, 3, 7, and 14 days after osteogenic differentiation were measured with quantitative real-time polymerase chain reaction (qRT-PCR). GAPDH was an internal control. **b** ALP levels in BM-MSCs on days 0, 3, 7, and 14 days after the osteogenic differentiation were quantified with enzyme-linked immunosorbent assay (ELISA). **c** BM-MSC osteogenic differentiation on days 0, 3, 7, and 14 was assessed with Alizarin Red Staining, under 100 × magnification. All the experiments have been performed in independent triplicate and experimental data were expressed as mean ± standard deviation (SD). **P < 0.01, ***P < 0.001, vs. 0 day. *FOXO3* Forkhead Box O3, *ALP* alkaline phosphatase, *BM-MSCs* bone marrow mesenchymal stem cells

To examine the role of FOXO3 in osteogenic differentiation of BM-MSCs, its expression in the BM-MSCs was measured on days 3, 7, and 14 after the osteogenic differentiation, and we found that FOXO3 expression was up-regulated in a time-dependent manner (Fig. 1a, *P* < 0.001), suggesting that FOXO3 may play a role in BM-MSC osteogenic differentiation.

As ALP is one of the key factors indicative of osteogenic differentiation [23], we subsequently measured ALP level in BM-MSCs on days 3, 7, and 14 after the osteogenic differentiation by ELISA. The results showed that the ALP level was also increased in a time-dependent manner (Fig. 1b, *P* < 0.001). Then, Alizarin Red Staining was employed to further confirm BM-MSC osteogenic differentiation. In Fig. 1c, microscope observation found that increases of red areas were after osteogenic differentiation for 3, 7, and 14 days.

### Effects of FOXO3 on the factors related to osteogenic differentiation of BM-MSCs

**Fig. 2:**
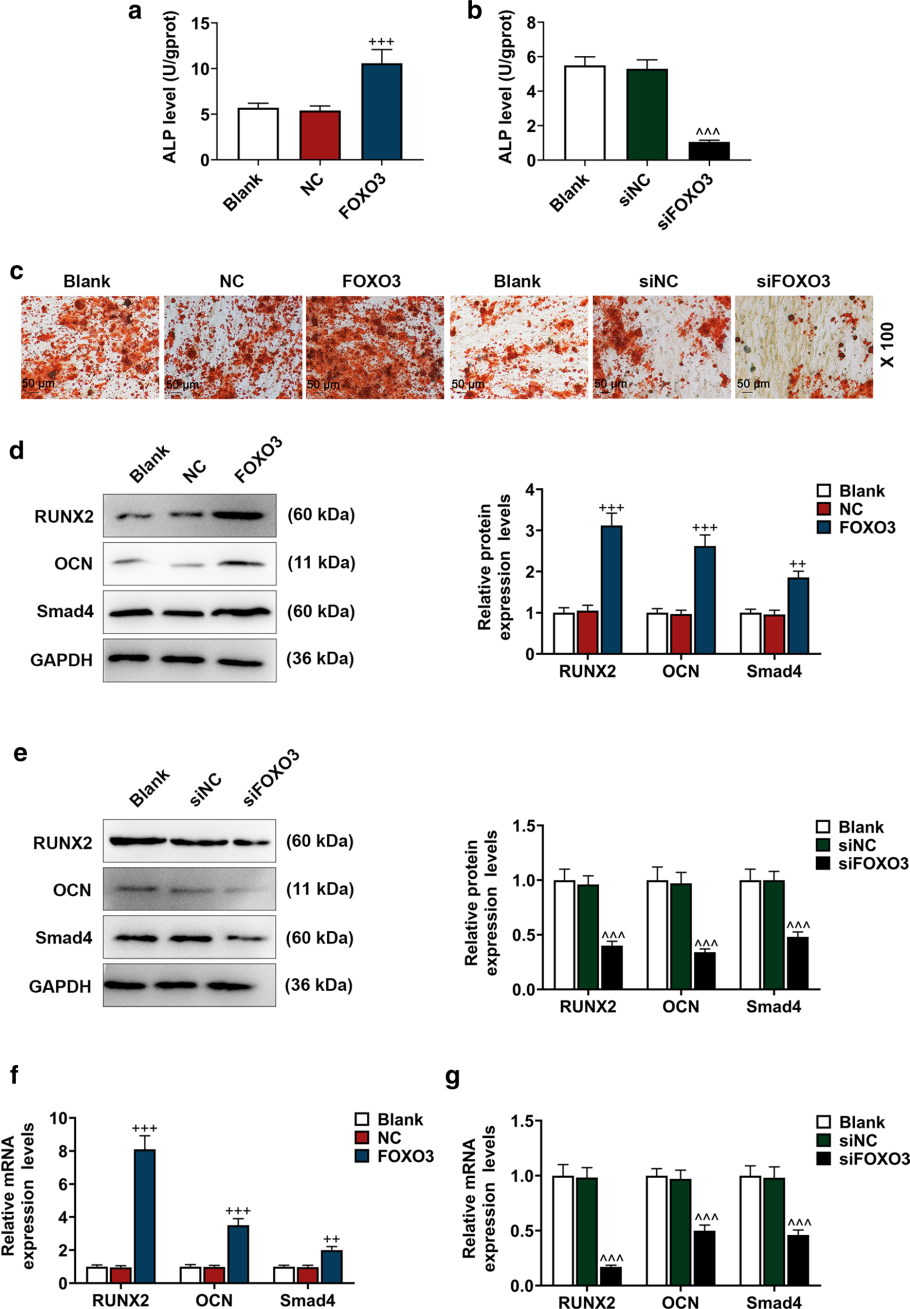
Effects of FOXO3 on BM-MSC osteogenic differentiation factors were detected. **a, b** ALP levels in BM-MSCs after up-regulating or down-regulating FOXO3 were quantified with ELISA. **c** Effects of up-regulating or down-regulating FOXO3 on BM-MSC osteogenic differentiation were detected with Alizarin Red Staining, under 100 × magnification. **d–g** Relative protein and mRNA expressions of factors related to osteogenic differentiation (RUNX2; OCN; Smad4) were measured with Western blot and qRT-PCR. GAPDH was an internal control. All the experiments have been performed in independent triplicate and the experimental data were expressed as mean ± standard deviation (SD). ^++^*P* < 0.01, ^+++^*P* < 0.001, vs. NC; ^^^^^*P* < 0.001, vs. siNC. *RUNX2* Runt-related transcription factor 2, *OCN* Osteocalcin, *Smad4* SMAD family member 4, *siNC* small interfering RNA for negative control

Then, to confirm the role of FOXO3 in osteogenic differentiation of BM-MSCs, we transfected lentivirus carriers of FOXO3 overexpression plasmids or siFOXO3 into the BM-MSCs and detected the effects of up-regulating and down-regulating FOXO3 expression on osteogenic differentiation of BM-MSCs. After the transfection of lentivirus carrier of FOXO3 overexpression plasmid, the ALP level in BM-MSCs was increased, and the transfection of lentivirus carrier of siFOXO3 caused an opposite result (Fig. 2a, b, *P* < 0.001). Alizarin Red Staining showed that there were more red areas after the transfection with lentivirus carrier of FOXO3 overexpression plasmid, but the opposite result was detected after transfection with lentivirus carrier of siFOXO3 (Fig. 2c).

RUNX2, OCN, and Smad4 are factors related to osteogenic differentiation [24], and their expressions after up-regulating or down-regulating FOXO3 were measured, and we found that the protein and mRNA expressions of RUNX2, OCN, and Smad4 were increased after the up-regulation of FOXO3 expression, whereas down-regulating FOXO3 resulted in opposite effects (Fig. 2d–g, *P* < 0.01). Therefore, it could be concluded that up-regulation of FOXO3 expression could promote osteogenic differentiation of BM-MSCs, whereas silencing of FOXO3 induced opposite results.

### Effects of FOXO3 on BM-MSC autophagy-related genes were detected

**Fig. 3:**
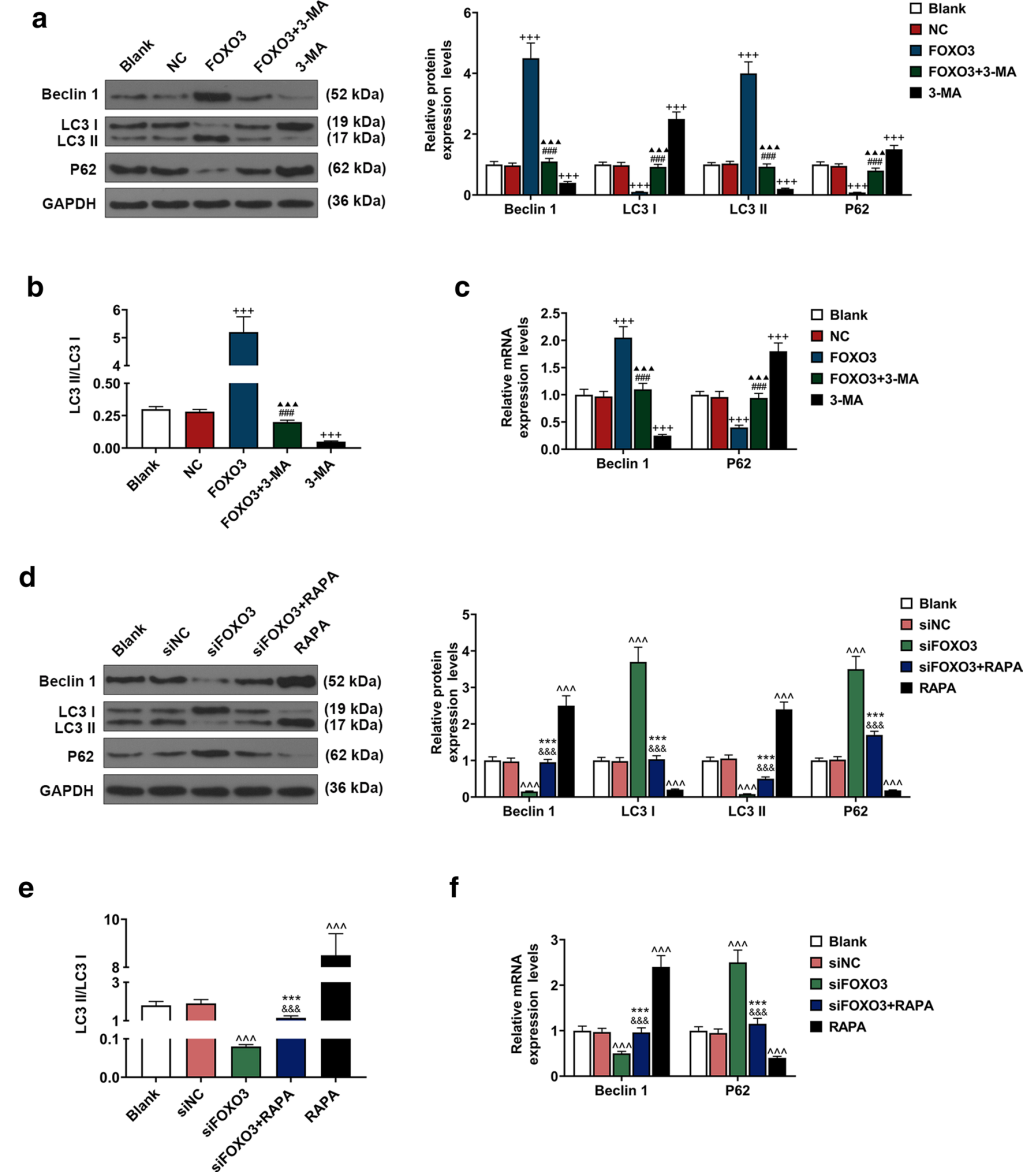
Effects of FOXO3 on gene expressions related to BM-MSC autophagy were detected. **a** Relative protein expressions of Beclin 1, LC3 I, LC3 II, and p62 after overexpressing FOXO3 and treatment with autophagy inhibitor 3-methyladenine (3-MA) were measured with Western blot. GAPDH was an internal control. **b** LC3 II/LC3 I ratio was calculated. **c** Relative protein expressions of Beclin 1 and p62 after overexpressing FOXO3 and 3-MA were measured with qRT-PCR. GAPDH was an internal control. **d** Relative protein expressions of Beclin 1, LC3 I, LC3 II, and p62 after siFOXO3 and rapamycin (RAPA) were measured with Western blot. GAPDH was an internal control. **e** LC3 II/LC3 I ratio was calculated. **f** Relative protein expressions of Beclin 1 and p62 after siFOXO3 and RAPA were measured with qRT-PCR. GAPDH was an internal control. All experiments have been performed in triplicate and experimental data were expressed as mean ± standard deviation (SD). ^+++^*P* < 0.001, vs. NC; ^###^*P* < 0.001, vs. FOXO3; ^▲▲▲^*P* < 0.001, vs. 3-MA; ^^^^^*P* < 0.001, vs. siNC; ^&&&^*P* < 0.001, vs. siFOXO3; ***P < 0.001, vs. RAPA. LC3: light chain 3

Beclin 1, LC3B, and p62 are related to cell autophagy [22]; therefore, to explore the effects of up-regulation or down-regulation of FOXO3 on BM-MSC autophagy, after up-regulating or down-regulating FOXO3 expression, the cells were treated either with autophagy inhibitor 3-MA or autophagy activator RAPA to determine the expressions of autophagy-related genes (Beclin 1, LC3 and p62). The data revealed that Beclin 1 and LC3 II expressions were down-regulated, while p62 and LC3 I expressions were up-regulated after the cells were treated with autophagy inhibitor 3-MA, whereas up-regulating FOXO3 expression resulted in opposite effects (Fig. 3a, c, *P* < 0.001). We also found that overexpressed FOXO3 reversed the effects of autophagy inhibitor 3-MA on the expressions of Beclin 1, LC3, and p62 (Fig. 3a, c, *P* < 0.001). Furthermore, we found a decrease of LC3 II/LC3 I ratio after treating the cells with 3-MA and an increase of LC3 II/LC3 I ratio after FOXO3 overexpression; moreover, overexpressed FOXO3 reversed the effects of 3-MA on the expressions of autophagy-related genes (Fig. 3b, *P* < 0.001).

As shown in Fig. 3d, f, the expressions of Beclin 1 and LC3 II were up-regulated, while LC3 I and p62 expressions were down-regulated after cells were treated with autophagy activator RAPA; however, up-regulating FOXO3 expression caused an opposite result (*P* < 0.001). In addition, silencing FOXO3 was found to reverse the effects of autophagy activator RAPA on the expressions of Beclin 1, LC3, and p62 (Fig. 3d, f, *P* < 0.001). Furthermore, we found an increase of LC3 II/LC3 I ratio after treating the cells with RAPA and a decrease of LC3 II/LC3 I ratio after silencing FOXO3, which also reversed the effects of PAPA on the expressions of autophagy-related genes (Fig. 3e, *P* < 0.001).

### FOXO3 promoted the osteogenic differentiation of BM-MSCs via enhancing autophagy

**Fig. 4:**
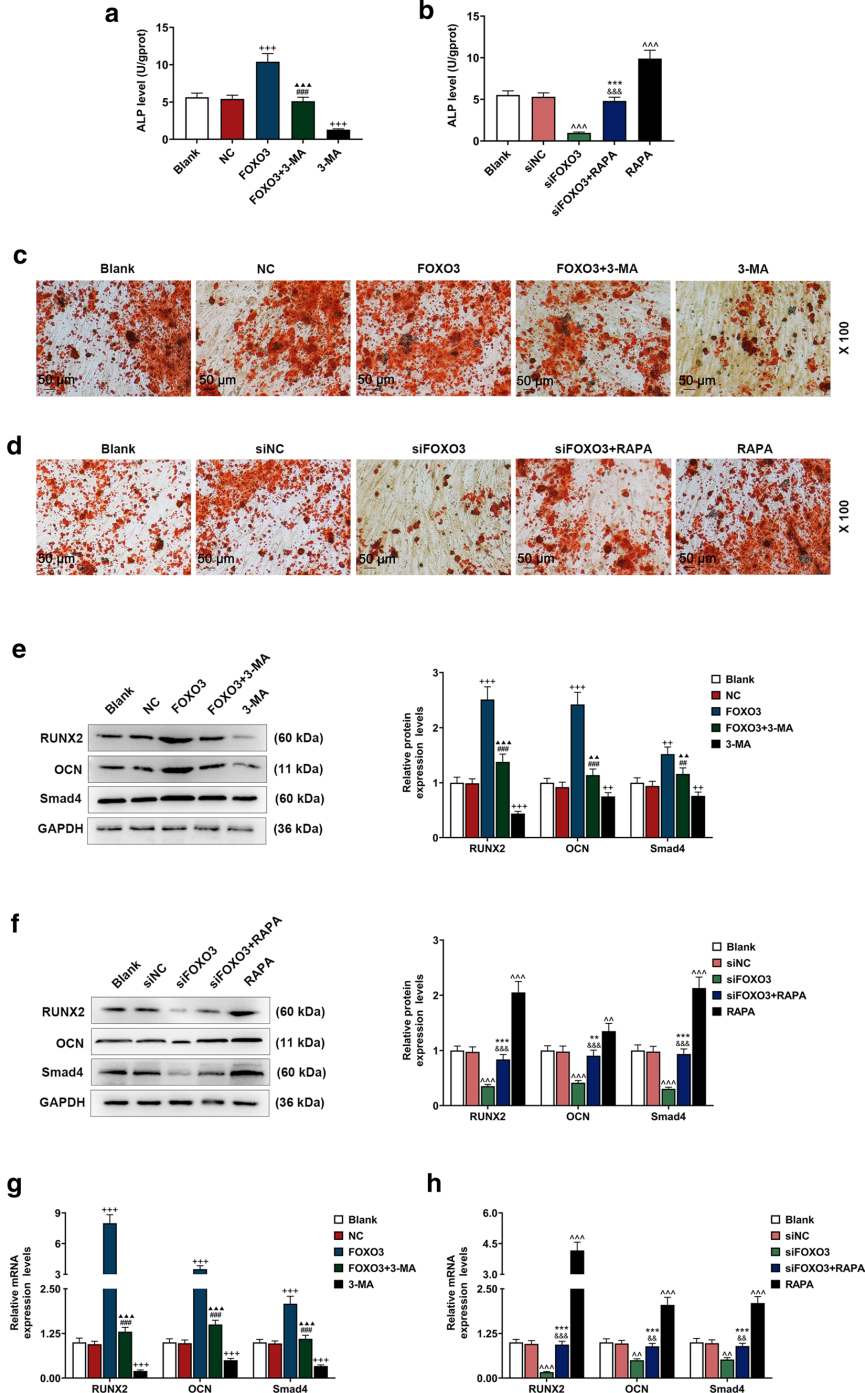
Effects of FOXO3 on BM-MSCs osteogenic differentiation via enhancing autophagy-related genes expressions were detected. **a, b** ALP levels in BM-MSCs after overexpressed FOXO3 and 3-MA treatment or siFOXO3 and RAPA treatment were quantified with ELISA. **c, d** Effects of overexpressed FOXO3 and 3-MA treatment or siFOXO3 and RAPA treatment on BM-MSCs osteogenic differentiation were detected with Alizarin Red Staining, under 100 × magnification. **e, h** Relative protein and mRNA expressions of factors related to osteogenic differentiation (RUNX2; OCN; Smad4) after overexpressing FOXO3 or siFOXO3 and 3-MA or RAPA treatment were measured with Western blot and qRT-PCR. GAPDH was an internal control. All experiments have been performed in triplicate and experimental data were expressed as mean ± standard deviation (SD). ^++^*P* < 0.01, ^+++^*P* < 0.001, vs. NC; ^##^*P* < 0.01, ^###^*P* < 0.001, vs. FOXO3; ^▲▲^*P* < 0.01,^▲▲▲^*P* < 0.001, vs. 3-MA; ^^^^^*P* < 0.001, vs. siNC; ^&&^*P* < 0.01, ^&&&^*P* < 0.001, vs. siFOXO3; **P < 0.01, ***P < 0.001, vs. RAPA

To uncover the relationship between osteogenic differentiation and autophagy of BM-MSCs, we measured ALP level and the gene expressions related to osteogenic differentiation after the transfection of lentivirus carriers for siFOXO3 or FOXO3 overexpression plasmids and treating cells with RAPA or 3-MA. We detected an increase of ALP level after the cell transfection with FOXO3 overexpression lentivirus carrier, whereas the transfection of siFOXO3 lentivirus carrier caused an opposite effect (Fig. 4a, b, *P* < 0.001). We also found that ALP level was decreased after treating the cells with 3-MA, whereas an opposite result was found following RAPA treatment (Fig. 4a, b, *P* < 0.001). Furthermore, FOXO3 overexpression reversed the effects of 3-MA on ALP level, and silencing of FOXO3 reversed the effects of RAPA (Fig. 4a, b, *P* < 0.001).

In Alizarin Red Staining, more red areas were detected after the cell transfection with overexpressed FOXO3 lentivirus carrier, but silencing FOXO3 resulted in an opposite effect (Fig. 4c, d). In addition, there were fewer red areas in 3-MA group, and more red areas in RAPA group (Fig. 4c, d). Besides, in FOXO3 + 3-MA group, the red areas were smaller than FOXO3 group but more than 3-MA group, whereas the red areas in siFOXO3 + RAPA group were more than siFOXO3 group but smaller than RAPA group (Fig. 4c, d).

The expressions of osteogenic differentiation-related genes (RUNX2; OCN; Smad4) were determined after transfecting lentivirus carriers for siFOXO3 or FOXO3 overexpression plasmids into the cells and treating the cells with RAPA or 3-MA. The results demonstrated that the above expressions were increased after overexpressed FOXO3 lentivirus carrier was transfected, but silencing FOXO3 resulted in an opposite effect (Fig. 4e–h, *P* < 0.01). Also, the expressions of RUNX2; OCN; Smad4 were up-regulated by RAPA treatment and down-regulated by 3-MA treatment (Fig. 4e–h, *P* < 0.01). Besides, FOXO3 reversed the effects of 3-MA on RUNX2, OCN, and Smad4 expressions, and silencing FOXO3 reversed the effects of RAPA on RUNX2, OCN, and Smad4 expressions (Fig. 4e–h, *P* < 0.001).

### FOXO3 was the target gene of miR-223-3p

**Fig. 5:**
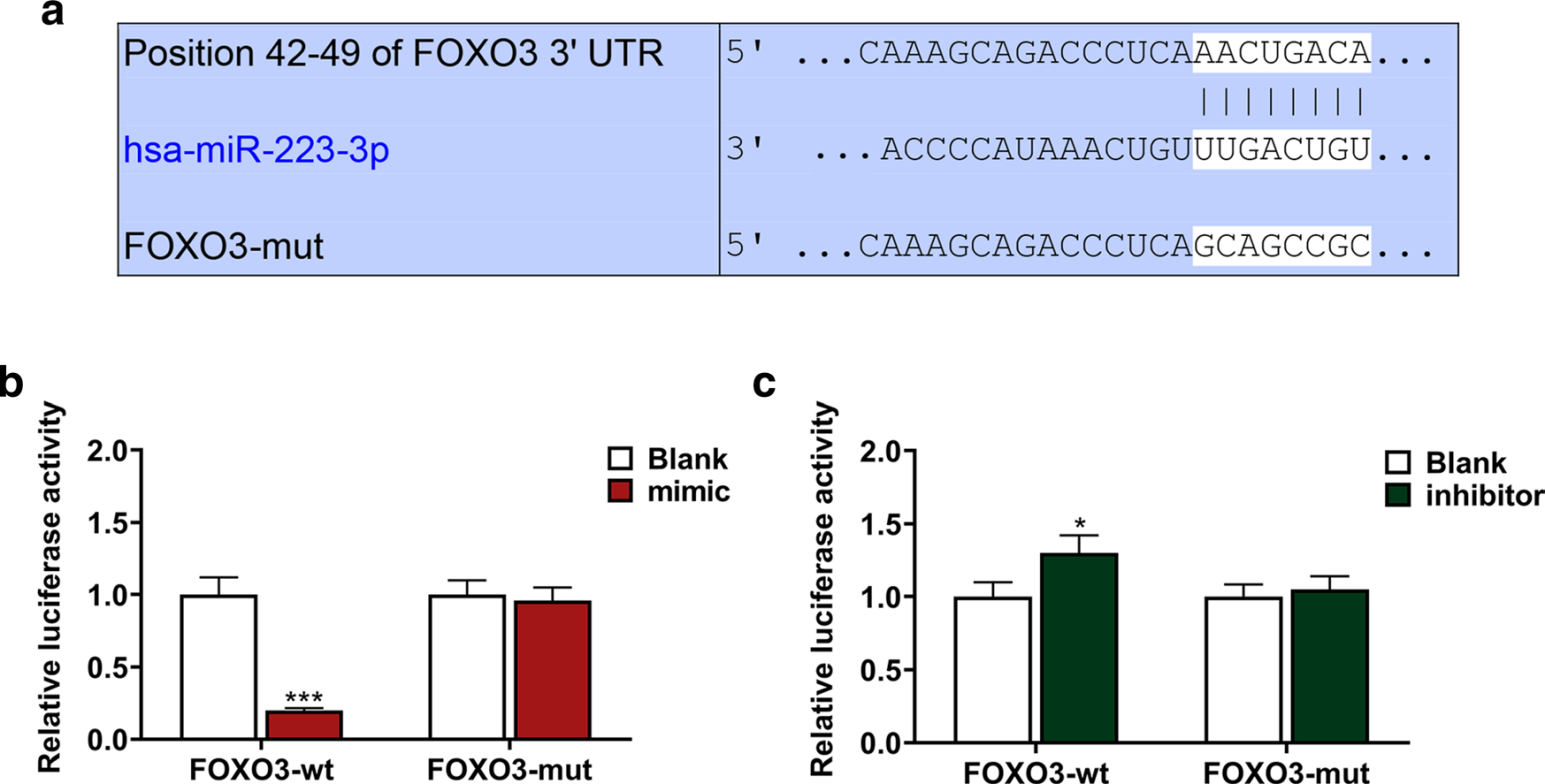
FOXO3 was the target gene for miR-223-3p. **a** Sequences of FOXO3-WT (top), miR-223-3p (middle), and FOXO3-MUT (below) were listed. **b, c** Dual-luciferase reporter assay showed that FOXO3 was the target for miR-223-3p. All experiments have been performed in independent triplicate and experimental data were expressed as mean ± standard deviation (SD). *P < 0.05, ***P < 0.001, vs. Blank. *WT* wild type, *MUT* mutated

By TargetScan 7.2, in our experiments, we successfully predicted and recognized FOXO3 as the target gene of miR-223-3p, with their mutual complementary binding sites in 3′-untranslated regions (3-UTRs; Fig. 5a). Then, to verify our discovery, dual-luciferase reporter assay was employed. It was found that in comparison with FOXO3-wt-Blank group, the luciferase activity in FOXO3-wt-mimic group was reduced, but in FOXO3-wt-inhibitor group, the luciferase activity was increased (Fig. 5b, c, *P* < 0.05). However, no significant difference was found in both FOXO3-mut-mimic and FOXO3-mut-inhibitor group in comparison with FOXO3-mut-Blank group. Therefore, it could be considered that FOXO3 was the target gene of miR-223-3p.

### Effects of FOXO3 targeted by miR-223-3p on gene expressions related to BM-MSCs’ autophagy

**Fig. 6:**
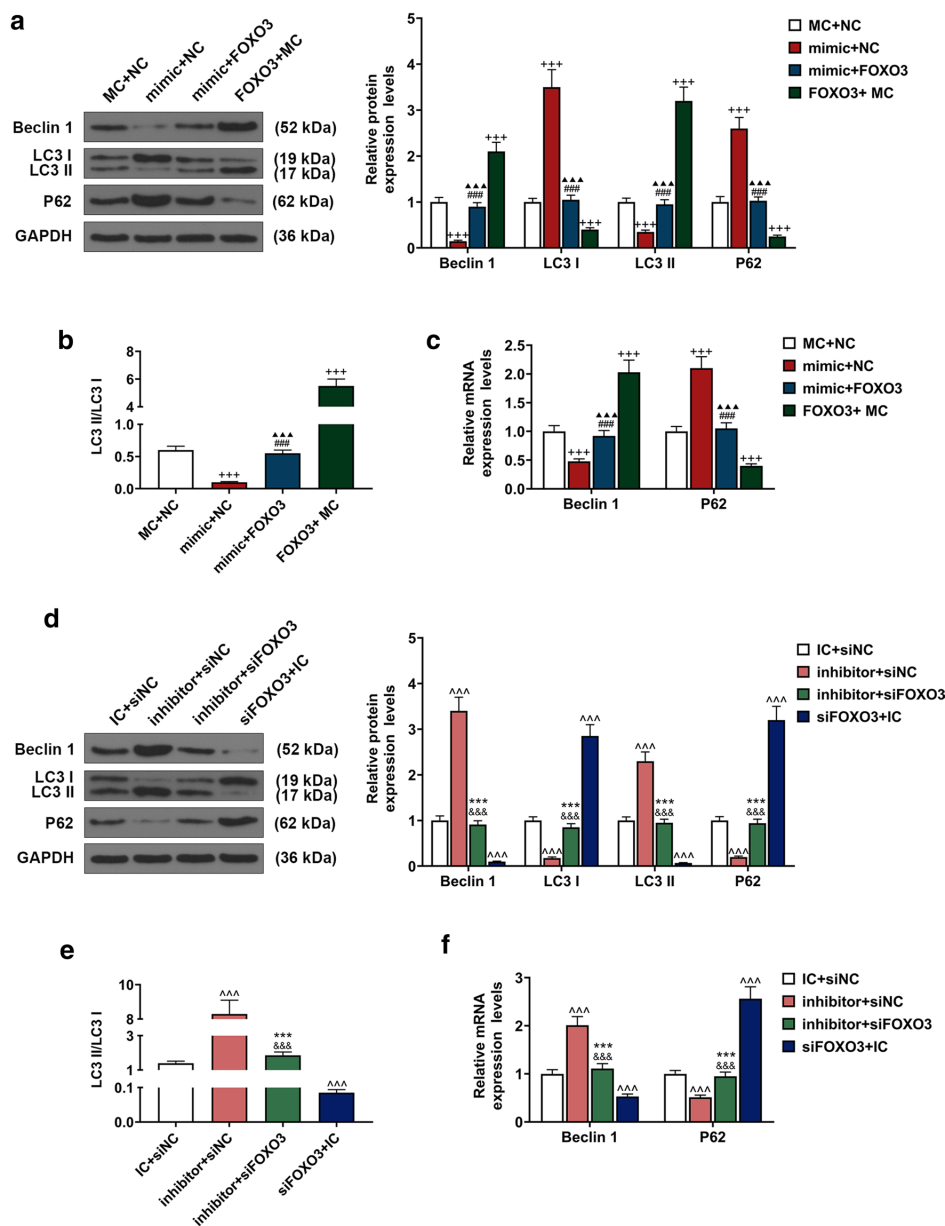
FOXO3 reversed the effects of miR-223-3p on autophagy-related gene expressions. **a** Relative protein expressions of Beclin 1, LC3 I, LC3 II, and p62 after overexpressing FOXO3 and up-regulating miR-223-3p were measured with Western blot. GAPDH was an internal control. **b** LC3 II/LC3 I ratio was calculated. **c** Relative protein expressions of Beclin 1 and p62 after overexpressing FOXO3 and up-regulating miR-223-3p were measured with qRT-PCR. GAPDH was an internal control. **d** Relative protein expressions of Beclin 1, LC3 I, LC3 II, and p62 after siFOXO3, and down-regulating miR-223-3p were measured with Western blot. GAPDH was an internal control. **e** LC3 II/LC3 I ratio was calculated. **f** Relative protein expressions of Beclin 1 and p62 after siFOXO3 and down-regulating miR-223-3p were measured with qRT-PCR. GAPDH was an internal control. All experiments have been performed in triplicate and experimental data were expressed as mean ± standard deviation (SD). ^+++^*P* < 0.001, vs. MC + NC; ^###^*P* < 0.001, vs. mimic + NC; ^▲▲▲^*P* < 0.001, vs. FOXO3 + MC; ^^^^^*P* < 0.001, vs. IC + siNC; ^&&&^*P* < 0.001, vs. inhibitor + siNC; ***P < 0.001, vs. siFOXO3 + IC. *MC* mimic control, *IC* inhibitor control

To examine the effects of miR-223-3p on BM-MSC autophagy and its correlation with FOXO3, we, respectively, transfected lentivirus carriers of miR-223-3p mimic, inhibitor, FOXO3 overexpression plasmid, and siFOXO3 into the BM-MSCs, and then measured gene expressions related to autophagy. It has been found that up-regulating miR-223-3p expression decreased Beclin 1 and LC3 II expressions, and increased LC3 I and p62 expressions (Fig. 6a, c, *P* < 0.001), whereas down-regulating miR-223-3p expression promoted Beclin 1 and LC3 II expressions and reduced LC3 I and p62 expressions (Fig. 6d, f, *P* < 0.001). Also, we found a reduced LC3 II/ LC3 I ratio after up-regulating miR-223-3p expression (Fig. 6b, *P* < 0.001), while down-regulating miR-223-3p expression caused an opposite result (Fig. 6e, *P* < 0.001). Therefore, it was concluded that up-regulating miR-223-3p expression could suppress the gene expressions related to BM-MSC autophagy, whereas down-regulating miR-223-3p resulted in an opposite effect.

Following the transfection of lentivirus carrier of overexpressed FOXO3 and siFOXO3, Beclin 1 and LC3 II expressions were increased, yet LC3 I and p62 expressions were reduced after FOXO3 overexpression plasmid transfection (Fig. 6a, c, *P* < 0.001). Silencing FOXO3, however, up-regulated LC3 I and p62 expressions, but down-regulated Beclin 1 and LC3 II expressions (Fig. 6a, c, *P* < 0.001). Meanwhile, we found an increase of LC3 II/ LC3 I ratio after FOXO3 overexpression (Fig. 6b, *P* < 0.001), and a reduction of LC3 II/ LC3 I ratio was detected after silencing FOXO3 (Fig. 6e, *P* < 0.001). In addition, we also found that overexpressed FOXO3 could reverse the effects of up-regulating miR-223-3p expression on autophagy-related gene expressions (Fig. 6a–c, *P* < 0.001), whereas silencing of FOXO3 reversed the effects of down-regulating miR-223-3p (Fig. 6d–f, *P* < 0.001).

### Effects of FOXO3 targeted by miR-223-3p on gene expressions related to osteogenic differentiation in BM-MSCs

**Fig. 7:**
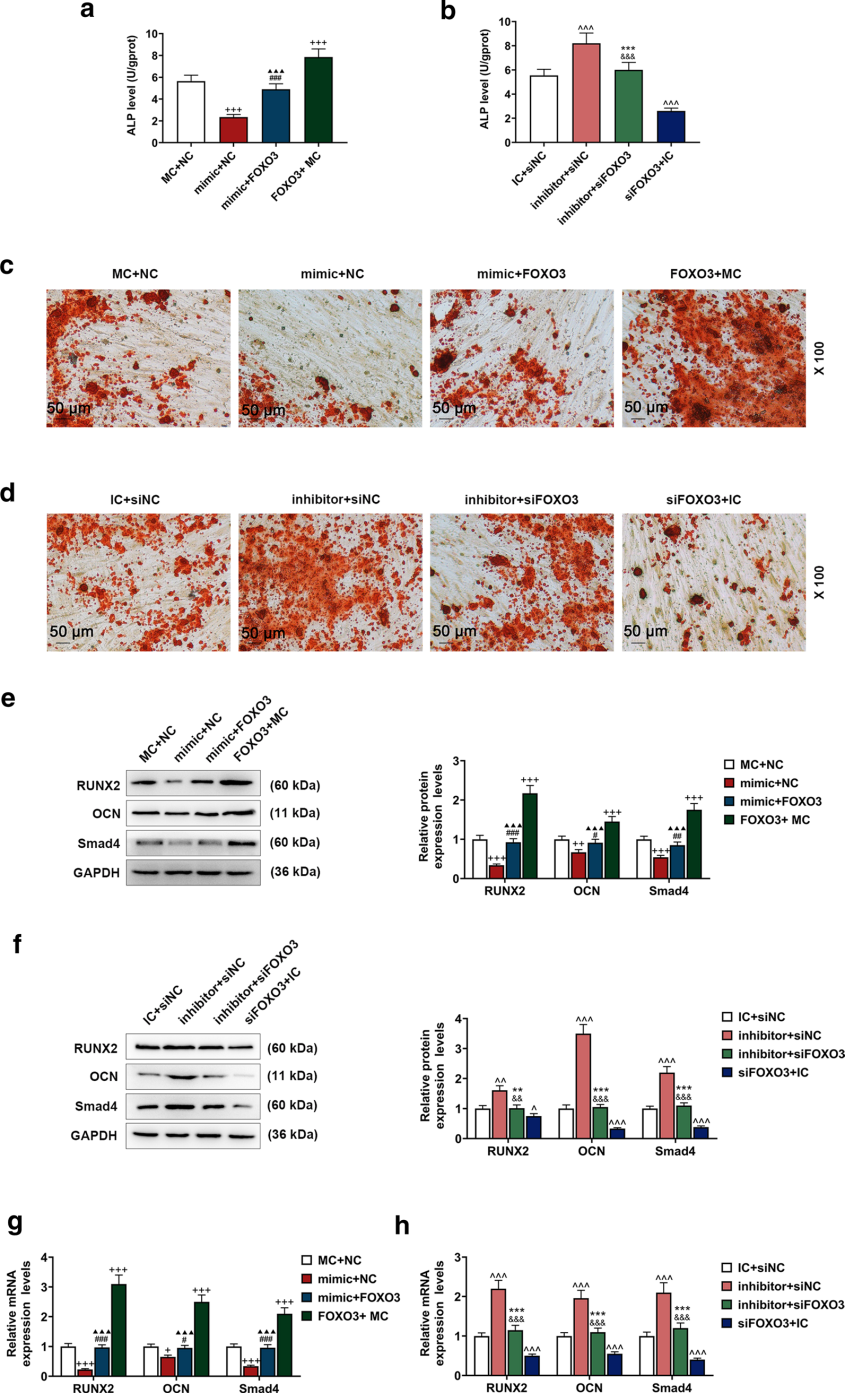
FOXO3 reversed the effects of miR-223-3p on BM-MSC osteogenic differentiation. **a, b** ALP levels in BM-MSCs after overexpressed FOXO3 or siFOXO3 and up-regulated or down-regulated miR-223-3p were quantified with ELISA. **c, d** Effects of overexpressed FOXO3 or siFOXO3 and up-regulated or down-regulated miR-223-3p on BM-MSC osteogenic differentiation were detected with Alizarin Red Staining, under 100 × magnification. **e–h** Relative protein and mRNA expressions of factors related to osteogenic differentiation (RUNX2; OCN; Smad4) after overexpressed FOXO3 or siFOXO3 and up-regulated or down-regulated miR-223-3p were measured with Western blot and qRT-PCR. GAPDH was an internal control. All experiments have been performed in triplicate and experimental data were expressed as mean ± standard deviation (SD). ^+^*P* < 0.05, ^++^*P* < 0.01, ^+++^*P* < 0.001, vs. NC; ^#^*P* < 0.05, ^##^*P* < 0.01, ^###^*P* < 0.001, vs. FOXO3; ^▲▲▲^*P* < 0.001, vs. 3-MA; ^^^*P* < 0.05, ^^^^*P* < 0.01, ^^^^^*P* < 0.001, vs. siNC; ^&&^*P* < 0.01, ^&&&^*P* < 0.001, vs. siFOXO3; ***P < 0.001, vs. RAPA

The ALP level and gene expressions related to osteogenic differentiation were determined, and we discovered that ALP level was reduced following the up-regulation of miR-223-3p expression (Fig. 7a, *P* < 0.001), whereas down-regulation of miR-223-3p expression up-regulated ALP level (Fig. 7b, *P* < 0.001). On the other hand, we found an increase of ALP level after the cell transfection of overexpressed FOXO3 plasmid, whereas silencing FOXO3 decreased ALP level (Fig. 7a, b, *P* < 0.001). Besides, overexpressed FOXO3 reversed the effects of up-regulating miR-223-3p expression on ALP level and silencing FOXO3 reversed the effects of down-regulating miR-223-3p on ALP level (Fig. 7a, b, *P* < 0.001).

Alizarin Red Staining showed reduced red areas following up-regulation of miR-223-3p expression, while down-regulation of miR-223-3p increased red areas (Fig. 7c, d). However, more red areas were found after the transfection of overexpressed FOXO3 lentivirus, while silencing FOXO3 reduced red areas (Fig. 7c, d). In addition, in mimic + FOXO3 group, the red areas were increased as compared with mimic + NC group, yet smaller red areas were detected in comparison with FOXO3 + MC group (Fig. 7c). A different result was observed in inhibitor + siFOXO3 group as compared with inhibitor + siNC group and siFOXO3 + IC group (Fig. 7d).

Then, we measured gene expressions related to osteogenic differentiation. It was found that after up-regulating miR-223-3p expression, protein, and mRNA expressions of genes related to osteogenic differentiation were down-regulated (Fig. 7e, g, *P* < 0.001), whereas down-regulating miR-223-3p expression caused an opposite effect (Fig. 7f, h, *P* < 0.001). Meanwhile, after the transfection of overexpressed FOXO3 lentivirus, osteogenic differentiation-related protein and mRNA expressions of genes were up-regulated, but silencing FOXO3 caused an opposite effect (Fig. 7e–h, *P* < 0.001). In addition, overexpression FOXO3 reversed the effects of up-regulating miR-223-3p expression on genes expressions related to osteogenic differentiation, whereas silencing FOXO3 reversed the effects of down-regulating miR-223-3p (Fig. 7e–h, *P* < 0.01).

## Discussion

FOXO family transcription factors play a pivotal role in several biological processes, including in cell proliferation, apoptosis, and differentiation [25]. FOXO3, a member of FOXO family, promotes osteogenic differentiation of MSCs by the regulation of redox homeostasis [6]. FOXO3a, a gene coding of FOXO3, has been confirmed as the target gene of miRNAs [26]. In our present study, we found FOXO3 that was the target gene of miR-223-3p, and its expression was up-regulated in a time-dependent manner during BM-MSCs’ osteogenic differentiation, showing that FOXO3 might play an important role in osteogenic differentiation of BM-MSCs. However, the detailed molecular mechanisms through which FOXO3 promoted BM-MSC osteogenic differentiation remains vague.

During BM-MSC osteogenic differentiation, the expressions of some related genes, including ALP, RUNX2, OCN, and Smad4, show up-regulated expressions [24]. ALP, produced by osteogenic cells such as osteoblasts, is one of the key regulators of osteogenic differentiation [23]. Runt-related transcription factor 2 (RUNX2) is associated with the phenotype of osteoblast and is also one of the key factors of transcription and of great importance in early osteogenic differentiation. Osteocalcin (OCN) is a mineralization regulator in the late stage of osteoblast development [27, 28]. Smad4, which acts as a common Smad, promotes RUNX-2-mediated osteogenic differentiation [29]. In our study, the expressions of ALP, RUNX2, OCN, and Smad4 were up-regulated after up-regulating FOXO3 in BM-MSCs, and overexpressed FOXO3 reversed such effects, suggesting that down-regulating miR-223-3p expression might promote the osteogenic differentiation of BM-MSCs via targeting FOXO3.

The previous studies also uncovered a relationship between autophagy and osteogenic differentiation in BM-MSCs [18]. Beclin 1, LC3, and p62 are autophagy-related proteins, and during autophagy, the expressions of Beclin 1, LC3 II, and p62 are up-regulated, but LC3 I expression is down-regulated [22]. Beclin 1, a gene of mammalian autophagy, plays an important role in the localization of autophagic proteins to a pre-autophagosomal structure (PAS) [30]. LC3 is ubiquitously distributed in mammalian tissues and cultured cells. During autophagy, cytosolic form of LC3 (LC3 I) is conjugated to phosphatidylethanolamine to form LC3-phosphatidylethanolamine conjugate (LC3 II) [31]. P62 is an adaptor protein that plays a vital role in selective autophagy [32]. Cheng et al. revealed a relation between autophagy and osteogenic differentiation and suggested that the activation of AMPK-dependent autophagy might contribute to osteogenic differentiation [16]. The previous study also pointed out that the role of FOXO3 in autophagy suggested that FOXO3 could control autophagy in skeletal muscle in vivo [33]. Consistently, we found that the gene expressions related to osteogenic differentiation were also up-regulated after activating autophagy of BM-MSCs, and that overexpressed FOXO3 reversed the effects of up-regulation of miR-223-3p on protein expressions of genes related to osteogenic differentiation and autophagy, which suggested that enhancing autophagy might be the mechanisms of BM-MSC osteogenic differentiation.

Some limitations of the present study should also be noted. We only examined the effects of miR-223-3p on BM-MSC osteogenic differentiation in vitro, its in vivo efficacy remained obscure and requires further studies.

In conclusion, our study was the first to illustrate the role of miR-223-3p in BM-MSC osteogenic differentiation with its effects on autophagy-related protein expressions. The current discovery of the role of miR-223-3p and its relation with FOXO3 in BM-MSCs improve the current understanding of the mechanisms underlying the osteogenic differentiation of BM-MSCs, and point out a potential clinical strategy for treatment of diseases related to abnormal osteogenic differentiation of BM-MSCs.
